# Supplementary figures and images for: Evaluation of a commercial AI-assisted cell counting software for dopaminergic neurons across species
Source: PLoS One. 2026 Mar 17;21(3):e0344621. doi: 10.1371/journal.pone.0344621 (PMC12994822; doi:10.1371/journal.pone.0344621)

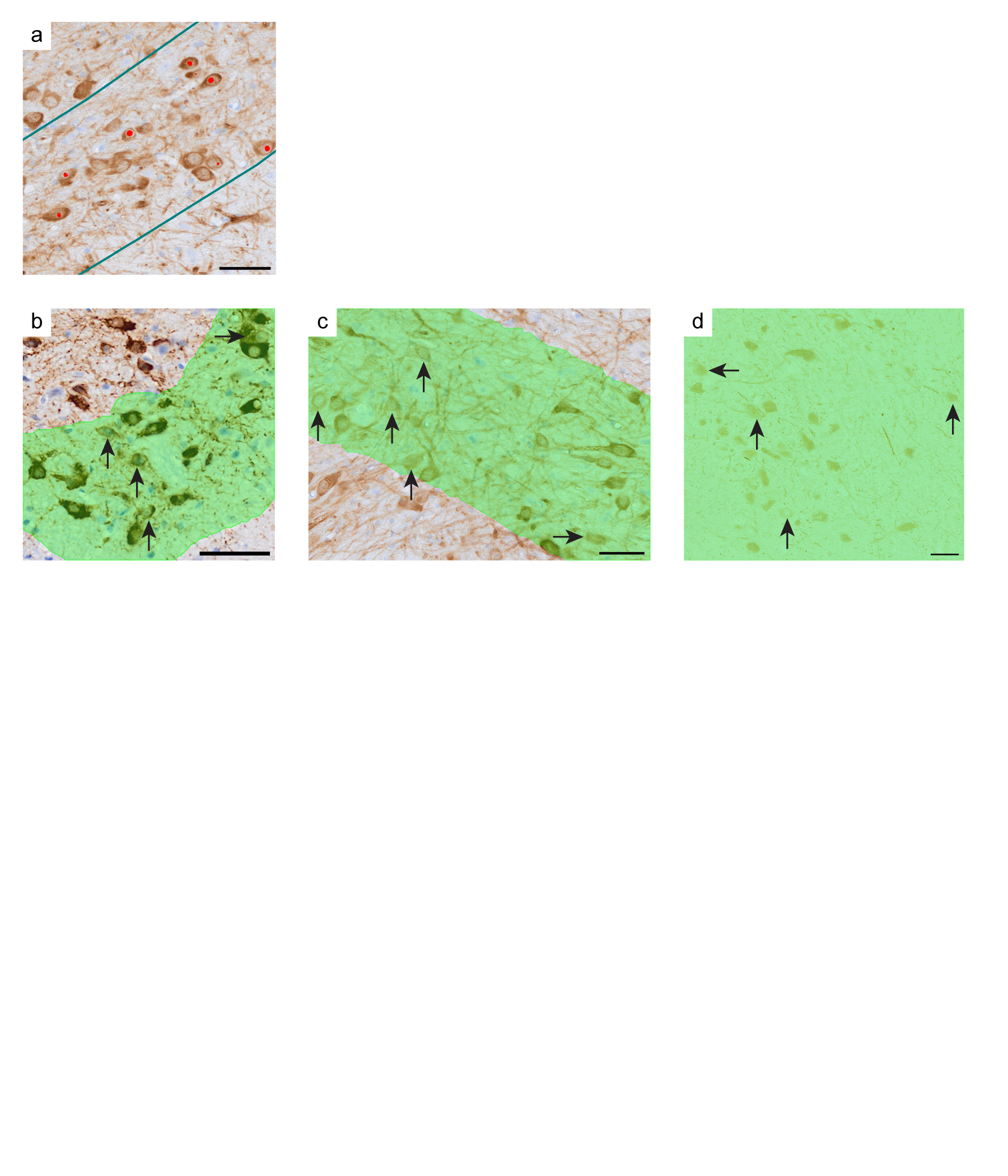

Supplement: S1 File — (ZIP) [file pone.0344621.s001.zip › Revised Figure S1.tif]

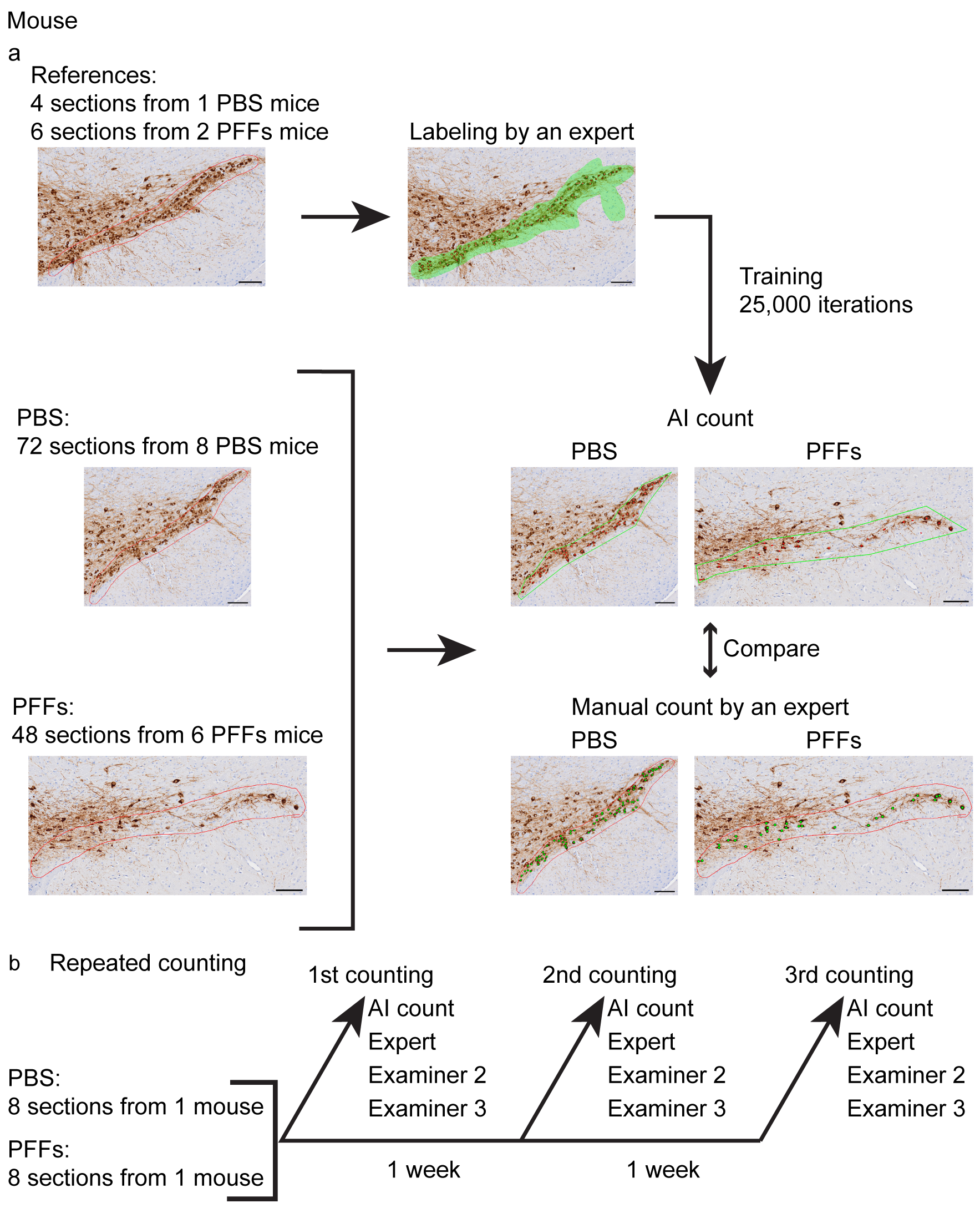

Supplement: S1 File — (ZIP) [file pone.0344621.s001.zip › Revised Figure S2.tif]

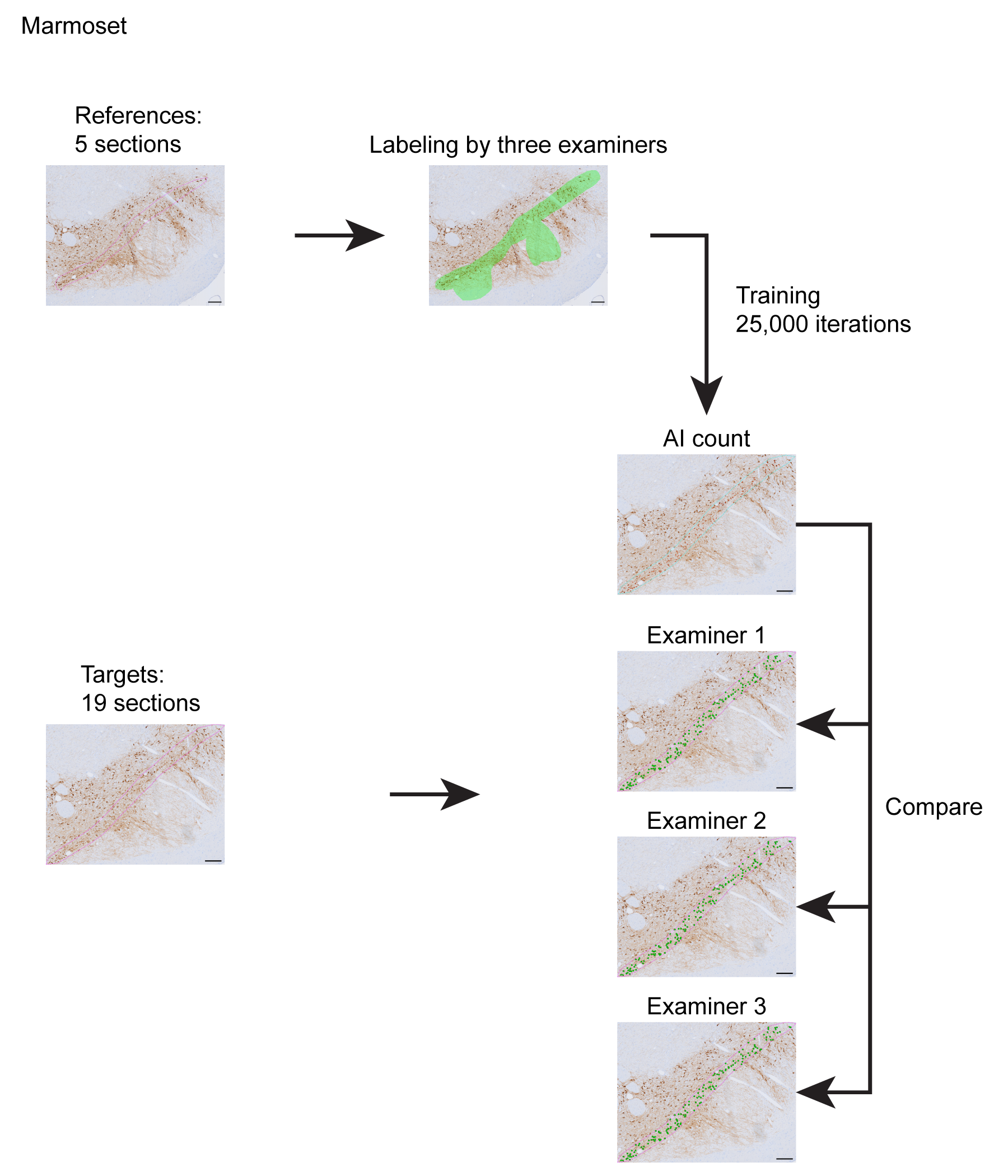

Supplement: S1 File — (ZIP) [file pone.0344621.s001.zip › Revised Figure S3.tif]

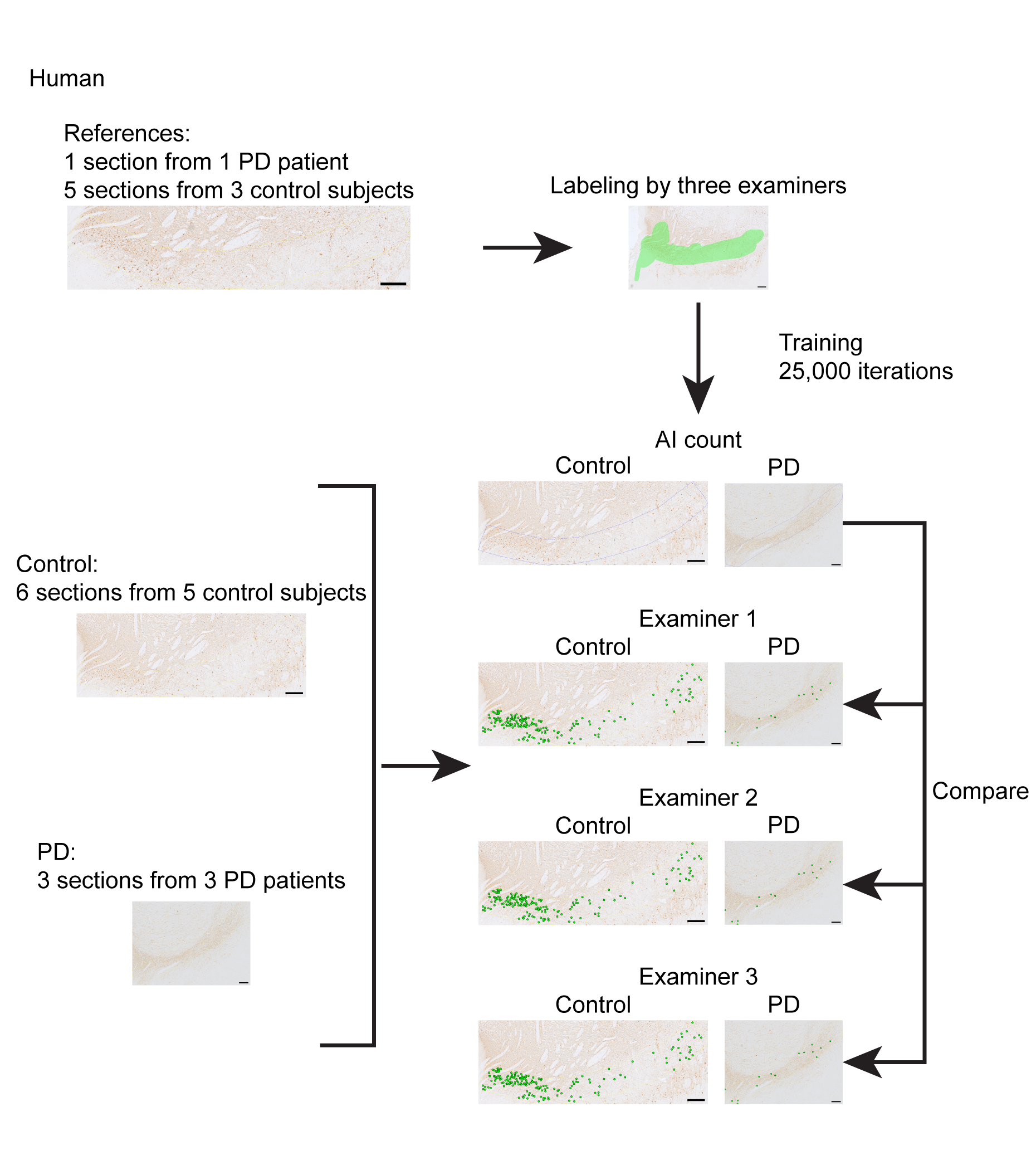

Supplement: S1 File — (ZIP) [file pone.0344621.s001.zip › Revised Figure S4.tif]
