# Supplementary material for: Evaluation of a commercial AI-assisted cell counting software for dopaminergic neurons across species
Source: PLoS One. 2026 Mar 17;21(3):e0344621. doi: 10.1371/journal.pone.0344621 (PMC12994822; doi:10.1371/journal.pone.0344621)
Supplement: Attachment — Submitted filename: Response to Reviewers.docx [file pone.0344621.s002.docx]

Reviewer #1: This manuscript addresses an important issue in Parkinson’s disease research: the reliable quantification of dopaminergic neurons in the substantia nigra pars compacta. The authors demonstrate that a commercially available CNN-based artificial intelligence system can quantify dopaminergic neurons with comparable accuracy to manual counting. Notably, the validation was carried out not only in mouse models but also in marmoset PD models and human PD brain samples, which is a significant strength and adds translational value to the work.
While the concept is interesting, several methodological and interpretational concerns should be clarified before the manuscript can be considered for publication.

1. Training images appear to include repeated use of the same specimens. In Supplementary Figures, it appears that for mouse and human samples, the same images (e.g., =3 or N=4) were used multiple times as teacher images. Could the authors clarify why the same specimens appear repeatedly? Clarifying these points is important because repetitive use of identical or highly similar images in a small dataset can lead to model overfitting, and therefore the reproducibility of the AI-based quantification method may be overestimated. In addition, how many individual dopaminergic neurons were used as ground-truth labels for training?

We appreciate the reviewer’s careful observation.

We would like to clarify that we used *distinct* images as reference (teacher) images for model training. In the Supplementary Figures, our intention was to demonstrate that multiple independent images were employed as reference datasets. However, we acknowledge that the current figure layout in which identical images were piled may have inadvertently given the impression that identical specimens were reused across training examples.

We apologize for this lack of clarity in presentation. These points have now been clarified in the revised manuscript and Supplementary Figures at the following locations:

- Lines 250-257 in the ***Mouse Samples*** section: Seventeen heterozygous A53T BAC-*SNCA* transgenic mice [10] were used in this study (9 PBS-treated and 8 PFF-treated mice). To confirm the genotype of the A53T BAC-*SNCA* transgenic mice, polymerase chain reaction was performed as previously described [10]. From these animals, a total of 130 sections were obtained. Of these sections, 4 sections from 1 PBS-treated and 6 sections from 2 PFF-treated mice were used as reference images, and the remaining 72 sections from 8 PBS-treated and 48 sections from 6 PFF-treated mice were used for cell counting. The ipsilateral SNcd corresponding to the PBS- or PFF-treated side was analyzed in each section.
- Supplementary Figure S2a

- Lines 288-292 in the ***Marmoset Samples*** section: Twenty-four sections from a two-year-old female marmoset (I5937; born at CLEA Japan, Inc.) that had received unilateral olfactory bulb injections of α-Syn PFFs were re-used from our previous study [10]. Of these sections, 5 were used as reference images, and the remaining 19 sections were used for cell counting. The bilateral SNcd were analyzed in each section.
- Supplementary Figure S3

- Lines 295-299 in the ***Human Samples*** section: Human samples were re-used from our previous study [31] and consisted of 4 sections from 4 patients with PD and 11 sections from 5 age-matched control subjects. Of these sections, 1 section from 1 PD patient and 5 sections from 3 controls were used as reference images, and the remaining 3 sections from 3 PD patients and 6 sections from 5 controls were used for cell counting. The unilateral SNc was analyzed in each section.
- Supplementary Figure S4

Regarding the ground-truth labels used for training, a total of 1,353; 1,313; and 1,398 individual dopaminergic neurons from mouse, marmoset, and human samples, respectively, were manually annotated and incorporated into the training dataset.

In the revised manuscript, we have clarified this explanation in the text.

- Lines 314-315: In total, 1,353 mouse, 1,313 marmoset, and 1,398 human dopaminergic neurons were annotated and incorporated into the training datasets.

2. Because TH-positive neuronal density can vary considerably along the rostro-caudal axis of the SNc, accurate quantification of the total number of dopaminergic neurons in the SNc generally requires unbiased stereological methods, or at least a comparison with the contralateral (non-lesioned) side to correct for section-to-section variability. In this study, it is not clearly described how the authors dealt with this issue.

We appreciate this insightful comment.

To address the reviewer’s concern, we would like to clarify our quantification strategy as follows:

1. Although we did not employ stereological methods, we systematically sampled the entire SNc by analyzing every 10th coronal section (80 µm apart), ensuring that the full rostro–caudal extent of the SNc was consistently included. This approach allowed us to evaluate the whole structure in a uniform and unbiased manner.
2. This systematic section-sampling method has been widely used and validated as a reliable way to quantify dopaminergic neuron numbers in several previous studies, and its reproducibility has been demonstrated (e.g., Luk et al., Science 2012; Taguchi et al., Brain 2020; Okuda et al., Neurotherapeutics 2022; Uemura et al., Nat Commun 2023).
3. In addition, full stereological analysis requires incorporation of section thickness and z-axis information, which is not compatible with our deep-learning–based pipeline. For this reason, we adopted the systematic sampling strategy described above, which is well accepted in the field and appropriate for automated image analysis.

In the revised manuscript, we have clarified this explanation in the text.

- Lines 276-278: Paraffinized brains were sectioned coronally in 8 µm thick on a microtome (EG1150, Leica Microsystems). For TH-positive cell counting, the entire SNc was sampled every 10th coronal section (80 µm apart), across its full rostro–caudal extent.

3. The authors state that AI-based counting was affected by staining quality, particularly in samples with weak TH staining. However, it is unclear whether such low-intensity images were included in the training set. Were teacher images selected to include a wide range of staining intensities, including weakly stained sections? Clarifying whether the training dataset captured realistic staining variability is critical for interpreting the robustness of the AI model in routine histopathological settings.

Thank you for this valuable comment. Low-intensity images were indeed included in the training dataset. We intentionally selected reference (teacher) images covering a wide range of TH staining intensities, from weak to strong signals, to ensure that the model could robustly handle variability in staining quality under realistic histopathological conditions.

In addition, images from PFF-treated mice and marmosets and PD patients were included so that the model could learn to detect morphologically distorted TH-positive neurons.

We have clarified this point explicitly in the revised manuscript and presented representative low-intensity reference images in Supplementary Figure 1b-d.

- Lines 213-216: Optimized immunohistochemical processes remain critical for reliable AI-based quantification, as staining intensity directly affects image contrast during whole-slide processing and leads to variability in AI counting results, even though the training dataset incorporated reference images spanning a wide range of staining intensities.
- Lines 316-321: Reference images were intentionally selected to cover a wide range of TH staining intensities, from weak to strong signals, to ensure robust performance under realistic histopathological conditions. Representative low-intensity regions of the reference images are shown in Supplementary Fig. S1b-d. In addition, images from PFF-treated mice and marmosets and PD patients were included to allow the model to learn morphological variations and distortions of TH-positive neurons associated with pathological conditions.
- Supplementary Figures S1b-d

- Lines 19-21 in legends for Supplementary Figures S1b-d: (b-d) Representative images with low-intensity TH-positive neurons (arrows) that were included in the training datasets for (b) mouse, (c) marmoset, and (d) human samples. Scale bar, 50 µm.

4. In the analysis of human specimens, neither AI-based nor manual counting detected a significant reduction in dopaminergic neurons in PD patients compared with healthy controls. Given that substantial neuronal loss in the SNc is a hallmark of PD pathology, this result requires further explanation. What is the authors’ interpretation of the absence of statistical significance?
Could this be due to sampling bias related to the anatomical level of the analyzed sections? As mentioned earlier, SNc neuron density varies across rostro–caudal levels. Given that the AI and manual methods both failed to show a difference, the issue may not be the counting algorithm itself, but rather the criteria used for section selection.

We appreciate this insightful comment. We believe that the absence of a statistically significant difference between PD patients and healthy controls—observed in both AI-based and manual counting—is primarily attributable to the limited number of available sections. This limited sampling likely introduced bias depending on the anatomical level analyzed along the rostro-caudal axis of the SNc.

In the revised manuscript, we have clarified this limitation and discussed that the lack of significance likely reflects restricted sampling coverage rather than a limitation of the counting algorithm itself.

- Lines 167-169: The analysis was based on a limited number of sections (three sections from 3 PD patients and six sections from 5 control subjects).
- Lines 233-238 in the **Limitations of the study** section: A limitation of the present study is the restricted number of human sections available for analysis, which resulted in limited sampling coverage along the rostro–caudal axis of the SNc. This constraint likely reduced the statistical power to detect neuronal loss in PD samples, despite the well-established pathology. Future studies using larger cohorts and anatomically systematic sampling will be required to fully validate the applicability of AI-based counting in human PD tissue.

5. The authors cite three prior studies that applied AI approaches to quantify dopaminergic neurons. However, to my knowledge, there is at least one additional report describing similar deep-learning–based approaches for TH-positive neuron detection (Chen et al. A user-friendly deep learning approach for robust dopaminergic neuron detection. Neurosci Lett. 2024, 836:137871. doi:10.1016/j.neulet.2024.137871.). The manuscript states that the present method performs with comparable inference accuracy, but it is not clearly discussed how this method differs from, or improves upon, those existing approaches.　What specific advantages does the proposed method (TruAI) offer over previously published models?

We appreciate the reviewer’s helpful suggestion and thank them for bringing our attention to the additional report by Chen et al. (2024). We have now included this study as an additional reference in the revised manuscript.

In our evaluation, TruAI demonstrated superior performance compared with one previously reported method (Kim et al., reference 25) and comparable inference accuracy to other published deep-learning–based approaches. Importantly, our validation was performed not only in mouse samples but also across marmoset and human specimens with substantially different cell sizes, staining conditions, and tissue characteristics, highlighting the generalizability of the approach.

We would like to clarify that TruAI itself is not a fundamentally novel deep-learning algorithm, but rather a commercially available, user-friendly platform designed to assist users in efficiently training neural networks to recognize user-defined targets. Similar deep-learning–based detection tasks can in principle be achieved using other frameworks, such as QuPath with appropriate plugins, as employed in the study by Chen et al., or other custom pipelines.

The primary advantage of TruAI lies in its accessibility and ease of use: it allows non-specialist users to perform AI-based neuron quantification without requiring programming expertise or the construction of custom training pipelines. This lowers the technical barrier for applying deep-learning–based cell counting in routine histopathological and experimental neuroscience settings.

Following the reviewer #2’s suggestion, we also expanded the literature review to include additional related work. Haghighi et al. (2023) proposed a self-supervised learning–based framework for TH-positive dopaminergic neuron soma segmentation and quantification and provided a publicly available annotated dataset, which is directly relevant to our task and has therefore been added to the manuscript and Table 3.

We have revised the manuscript to clarify these points, to explicitly distinguish platform-level advantages from algorithmic novelty, and to include the additional citations suggested by the reviewers.

- Lines 74-76: To our knowledge, multiple CNN-based AIs have been previously reported for enumerating TH-positive cells in the SNc of neurotoxic PD mouse models [23-27]; however, they are yet to be recognized as the gold standard.
- Lines 184-212 in the **Discussion** section: To our knowledge, multiple deep-learning–based approaches have been reported for automated quantification of TH-positive dopaminergic neurons in the mouse SNc [23–27]. The performance metrics of these models are summarized in Table 3. One of these models exhibited 69.0% precision, 79.3% recall, and 73.0% F1-score, which were slightly lower than those of the present study [25]. The remaining models showed high performance, with precision ranging from 86.0–95.3%, recall from 87.8–95.5%, and F1-scores from 88.0–95.3% [23,24,26,27]. These results indicate that CNN-based AI enables automated TH-positive cell counting, which is difficult to achieve using classical image-processing approaches. Most previously reported models were primarily evaluated using neurotoxic PD models, such as 6-hydroxydopamine (6-OHDA) [23] or 1-methyl-4-phenyl-1,2,3,6-tetrahydropyridine (MPTP) models [24,25], which are known to induce large, dose-dependent reductions in TH-positive neurons (approximately 50–90%) [32,33]. More recently, Chen et al. demonstrated a deep-learning–based quantification pipeline validated in both neurotoxic (MPTP) and PFF models [27], indicating that CNN-based approaches can be applied across distinct PD paradigms. In addition, Haghighi et al. also reported a CNN-based quantification framework applied to PD model mice [26], although the specific PD induction method was not explicitly categorized. Notably, α-synuclein PFF models are generally characterized by more gradual and modest reductions in TH-positive neurons compared with neurotoxic models. The present study focused on the α-syn PFFs model, which has been reported to show an approximately 38% decrease in TH-positive cells at 2 months post-injection [34]. Our results demonstrate that the proposed AI-based approach can reliably quantify TH-positive neurons even under conditions of relatively small neuronal loss. Taken together, these findings indicate that the method implemented in this study achieves performance comparable to previously reported CNN-based approaches while being applicable to PD models with varying degrees of neuronal loss. In addition, by leveraging a commercially available, user-friendly AI platform, our approach facilitates efficient training and application of deep-learning–based neuron quantification without requiring specialized programming expertise, thereby lowering the technical barrier for routine use in experimental neuroscience and histopathological analysis.
- **Table 3.** Performance metrics of CNN-based AIs for counting TH-positive cells reported to date.

| **Report** | **Precision** | **Recall** | **F1-score** |
| --- | --- | --- | --- |
| Penttinen AM et al., 2018 | 88.5% | 87.8% | 88.2% |
| Zhao S et al., 2018 | 95.0% | 91.9% | 93.4% |
| Kim D et al., 2023 | 69.0% | 79.3% | 73.0% |
| Haghighi et al., 2023 | 95.3% | 95.5% | 95.3% |
| Chen et al., 2024 | 86.0% | 91.0% | 88.0% |

Reviewer #2: The authors present an interesting manuscript that highlights the use of the commercial AI-assisted cell counting software, TruAI, developed by Olympus, in dopaminergic neuron counting. The study investigates the utility of this software across three use cases—Parkinson’s disease (PD) animal models with PFF injections, marmoset models with PFF injections, and human PD samples. While the findings provide valuable insights for the broader scientific community, the study would benefit from further rigor, expanded analysis, and coherence to strengthen its conclusions. Below are specific points for consideration:

1. Comments on the Mouse PFF Model:

PFF Injection Duration:
The manuscript does not clearly state the duration of PFF injections. This information is critical to understanding the timeline for induced pathology and assessing the reliability of the reported tyrosine hydroxylase (TH) loss. Please provide explicit details about the injection schedule and post-injection timepoints.

We apologize for the lack of clarity regarding the PFF injection schedule. In this study, PFFs were injected into the striatum of 3-month-old female mice, and pathological evaluations were performed 2 months after the injection.

- Lines 267-271: As previously reported [34], 3-month-old mice were anesthetized with isoflurane and then stereotaxically inoculated with 2 µl of α-syn PFFs or PBS into the unilateral dorsal striatum (coordinates: Anterior-Posterior +0.2 mm relative to the bregma, Medial-Lateral +2.0 mm from the midline, Dorsal-Ventral -2.6 mm from the skull surface) using a 33-gauge Neuros syringe (Hamilton company).
- Lines 274-276: Two months after the injection of PFFs or PBS, mice were anesthetized with sevoflurane and transcardially perfused with cold PBS followed by 4% (w/v) paraformaldehyde (PFA) in PBS at 4°C overnight.

Phospho-synuclein Staining:
To confirm the presence of synuclein pathology caused by the PFF injections, phospho-synuclein staining should be performed and shown in the results. Without such confirmation, it is difficult to ascertain that the observed changes in TH cells are the result of α-synuclein pathology.

Thank you for pointing this out. We performed phospho-synuclein staining using an antibody against phosphorylated α-synuclein to verify the presence of α-synuclein pathology induced by PFF injections. The results are presented in Figures 1a-d. These data confirm that the pathological accumulation of phosphorylated α-synuclein occurred as expected.

- Lines 87-92: As previously reported [29], phosphate-buffered saline (PBS) or PFFs were administered to the unilateral striatum of bacterial artificial chromosome (BAC) transgenic mice expressing familial PD-linked A53T mutant human α-syn (A53T BAC-*SNCA* transgenic mice), and dopaminergic neurons in the ipsilateral substantia nigra were evaluated with immunohistochemistry using anti p-α-syn. Compared with the PBS group (Fig. 1a, b), the PFFs group exhibited p-α-syn aggregates in the SNc (Fig. 1c, d).
- Figures 1a-d

Sampling and Section Analysis:
The authors use 8 μm thick tissue sections and analyze only one section per mouse (10 sections from 10 mice). This limited sampling is insufficient for assessing the substantia nigra (SN), where TH+ neurons may vary greatly from section to section due to technical artifacts and the topology of the SN.

Although the authors mention that they captured images from many additional sections, they did not analyze these. This represents a missed opportunity to provide a more comprehensive analysis.

To justify claiming TH loss in the PFF mouse model, the authors need to evaluate the entire SN or at least perform a more systematic analysis across different rostro-caudal levels. As it stands, the authors can only claim to have counted TH cells in a limited number of sections.

Thank you for raising this important point regarding sampling and section analysis.

To address the reviewer’s concern about systematic evaluation of the SNc, we would like to clarify our quantification strategy as follows:

1. We sampled the entire SNc by analyzing every 10th coronal section (80 µm apart) across its full rostro–caudal extent. This approach allowed uniform and unbiased evaluation of the entire structure, rather than relying on a single representative section.
2. This systematic sampling strategy has been widely used and validated for quantifying dopaminergic neuron numbers in the SNc, and its reproducibility has been demonstrated in multiple previous studies (e.g., Luk et al., Science 2012; Taguchi et al., Brain 2020; Okuda et al., Neurotherapeutics 2022; Uemura et al., Nat Commun 2023).
3. We acknowledge that full stereological analysis incorporates section thickness and z-axis information; however, such approaches are not compatible with our deep-learning–based image analysis pipeline, which relies on two-dimensional whole-slide images. Therefore, we adopted the systematic section-sampling method described above, which is well accepted in the field and appropriate for automated image-based quantification.

In the revised manuscript, we have clarified this explanation in the text.

- Lines 276-278: Paraffinized brains were sectioned coronally in 8 µm thick on a microtome (EG1150, Leica Microsystems). For TH-positive cell counting, the entire SNc was sampled every 10th coronal section (80 µm apart) across its full rostro–caudal extent.

The issue of sample size and the definition of data points is addressed in detail in our response to the following comment, “Discrepancies in Sample Details.”

Discrepancies in Sample Details:
There are inconsistencies between the sample details in the Methods section and the data presented in figures. For instance, the Methods state “10 mice (4 PBS and 6 PFFs), 5 marmosets, and 6 human (1 PD and 5 controls),” but figures show a higher number of data points than described. Please clarify and align the information throughout the manuscript.

We appreciate the reviewer’s careful observation regarding the apparent discrepancies in sample numbers and apologize for the lack of clarity in the original presentation. The confusion arose because the number of animals, the number of sections, and the number of data points shown in the figures were not sufficiently distinguished.

Seventeen heterozygous A53T BAC-*SNCA* transgenic mice were used in this study (9 PBS-treated and 8 PFF-treated mice). From these animals, a total of 130 sections were obtained. Of these sections, 4 sections from 1 PBS-treated and 6 sections from PFFs-treated mice were used as reference images, and the remaining 72 sections from 8 PBS-treated and 48 sections from 6 PFFs-treated mice were used for cell counting. Each data point in Figures 1i-k represents the TH-positive cell count per animal.

Twenty-four sections from a marmoset were used in this study. Of these sections, 5 were used as reference images, and the remaining 19 sections were used for cell counting. 38 ROIs corresponding to the bilateral SNcd of 19 sections from a single animal were analyzed. Each data point in Figures 2e-h represents the TH-positive cell count per ROI.

Four sections from 4 patients with PD and 11 sections from 5 control subjects were used in this study. Of these sections, 1 section from 1 PD patient and 5 sections from 3 controls were used as reference images, and the remaining 3 sections from PD patients and 6 sections from 5 controls were used for cell counting. Each data point represents the TH-positive cell count per ROI in Figures 3e-g and the TH-positive cell density per ROI in Figure 3h.

In the revised manuscript, we have clarified these points in the Mouse, Marmoset, and Human Samples sections and in the legends for Figures 1i-k, 2e-h, 3e-h, and have reorganized Supplementary Figures S2-4 to avoid potential misunderstanding.

- Lines 250-257 in the ***Mouse Samples*** section: Seventeen heterozygous A53T BAC-*SNCA* transgenic mice [10] were used in this study (9 PBS-treated and 8 PFF-treated mice). To confirm the genotype of the A53T BAC-*SNCA* transgenic mice, polymerase chain reaction was performed as previously described [10]. From these animals, a total of 130 sections were obtained. Of these sections, 4 sections from 1 PBS-treated and 6 sections from 2 PFF-treated mice were used as reference images, and the remaining 72 sections from 8 PBS-treated and 48 sections from 6 PFF-treated mice were used for cell counting. The ipsilateral SNcd corresponding to the PBS- or PFF-treated side was analyzed in each section.
- Lines 288-292 in the ***Marmoset Samples*** section: Twenty-four sections from a two-year-old female marmoset (I5937; born at CLEA Japan, Inc.) that had received unilateral olfactory bulb injections of α-Syn PFFs were re-used from our previous study [10]. Of these sections, 5 were used as reference images, and the remaining 19 sections were used for cell counting. The bilateral SNcd were analyzed in each section.
- Lines 295-299 in the ***Human Samples*** section: Human samples were re-used from our previous study [31] and consisted of 4 sections from 4 patients with PD and 11 sections from 5 age-matched control subjects. Of these sections, 1 section from 1 PD patient and 5 sections from 3 controls were used as reference images, and the remaining 3 sections from 3 PD patients and 6 sections from 5 controls were used for cell counting. The unilateral SNc was analyzed in each section.

2. Technical Questions on the Software and Methodology:

Effect of Signal Contrast:
The authors state that signal contrast impacts the accuracy of AI-based detection. Please provide specific details on:
How signal contrast was manipulated.
The magnitude of changes in contrast that led to failure in detection.
The percentage of counting errors introduced by differences in contrast.

We appreciate the reviewer’s request for clarification regarding the effect of signal contrast on the accuracy of AI-based detection.

Signal contrast was not artificially manipulated in this study. Instead, we evaluated its impact by testing two different anti-TH antibodies during preliminary experiments. Based on staining quality and signal-to-noise ratio, we selected the antibody that provided superior contrast for all subsequent analyses. In addition, image acquisition parameters (e.g., exposure and gain) were appropriately adjusted to optimize signal contrast under each staining condition.

Representative examples illustrating the magnitude of contrast differences that led to detection failure are shown in Figures 1l and 1m. These images were stained with the antibody that produced weaker contrast (Millipore, #AB152, rabbit, 1:800 dilution).

In this section, AI-based counting detected 27 TH-positive neurons (marked in red), whereas manual counting identified 43 neurons (marked in green, True positive). No false-positive detections were observed. This corresponds to a false-negative rate of 37.2%, attributable to reduced signal contrast. These results highlight that insufficient signal contrast can substantially impair the performance of AI-based detection.

- AI-based counting for weak staining sample (scale bar, 50 µm)

- Manual counting for weak staining sample (scale bar, 50 µm)

Experience of the Testers:
Please elaborate on the qualifications and experience of the “experienced testers” who were responsible for validating the AI-based counts. Information about their expertise in histology, microscopy, or AI-assisted analysis will help gauge the reliability of their findings.

The tester is a board-certified neurologist specializing in Parkinson’s disease, with 13 years of clinical experience. The tester also has eight years of extensive research experience with Parkinson’s disease model mice. This includes comprehensive expertise in neuropathological evaluation of PD models, encompassing detailed analysis of α-synuclein–related pathology and highly accurate quantification of dopaminergic neuronal loss. In addition, the tester is well trained in biochemical assays and behavioral analyses relevant to PD pathogenesis. The tester has also served as the first author on peer-reviewed publications involving pathological characterization of Parkinson’s disease mouse models ([doi.org/10.1093/brain/awz380](https://doi.org/10.1093/brain/awz380)).

- Lines 337-340: The expert tester (T.T.) is a board-certified neurologist specializing in Parkinson’s disease, with 13 years of clinical experience and over eight years of research experience in Parkinson’s disease model mice, including neuropathological evaluation and accurate quantification of dopaminergic neuronal loss [10].

Segmentation of the Substantia Nigra (SNpc):
The images provided show a diffuse segmentation of the SNpc by the AI algorithm. How was the manual segmentation of the SNpc aligned with the AI algorithm’s segmentation? If there were discrepancies between the two methods, how were these addressed, and how do they impact the reported cell counts?

We appreciate the reviewer’s insightful comment. As the primary focus of this study was to evaluate the performance of AI-based cell counting, segmentation of the substantia nigra pars compacta (SNpc) was performed manually rather than by the AI algorithm.

We manually generated the same regions of interest (ROIs) for AI-based counting and manual counting.

- Lines 309-310: ROI segmentation was manually performed by an expert for mouse samples and by three non-experts for marmoset and human.

Handling of Overlapping/Dense TH+ Cells:
The TH counting algorithm seems to rely on the nuclei of individual TH cells for detection. How does the software resolve overlapping or dense TH+ cells, which are common in the SNpc and often introduce challenges in automated analysis? The representative images provided do not appear to depict such scenarios. Please discuss and provide evidence that TruAI can handle these situations, or clarify the limitations of the software in this regard.

We thank the reviewer for raising this important point regarding densely packed or overlapping TH-positive neurons in the SNpc. Reference image used for TruAI included sections containing overlapping and high-density TH-positive cells, as illustrated in the representative example provided below (not shown in the manuscript). The deep learning model was trained to recognize individual cells based on features learned from these reference datasets.

- Reference image with containing overlapping TH-positive cells (scale bar, 50 µm)

Importantly, TruAI does not employ a rule-based algorithm specifically designed to separate densely packed cells. Instead, its performance relies on pattern recognition learned from the reference images used during training, enabling the model to distinguish the nuclei of individual TH-positive cells even in regions where TH-positive cells overlap. Nevertheless, we acknowledge that under conditions of extreme cell crowding, detection accuracy may be reduced. This limitation is now explicitly described in the revised manuscript as a technical constraint of the current implementation of TruAI.

- Lines 239-246 in the **Limitation of the study** section: Another limitation relates to the performance of TruAI in regions with extreme cell density or severe overlap of TH-positive neurons. TruAI relies on pattern recognition learned from reference images rather than explicit rule-based separation of densely packed cells; therefore, detection accuracy in such regions depends on whether similar patterns were sufficiently represented in the training dataset. In the present study, no regions exhibiting extreme cellular crowding that would compromise reliable cell identification were observed. Nevertheless, under conditions of unusually high cell density, detection accuracy may be reduced, and careful visual validation remains important.

3. Clarifications on Results and References:

Line 166:
The statement regarding a “38% decrease in 2 months” is unclear. Does this pertain to 2 months post-PFF injection, or is it referring to 2-month-old animals? Please clarify and provide appropriate citations to support this claim.

Thank you for pointing this out. It is referring to 2 months post-PFF injection (Okuda S, et al., reference 34), and we have clarified this point in the revised manuscript.

- Lines 202-204: The present study focused on the α-syn PFFs model, which has been reported to show an approximately 38% decrease in TH-positive cells at 2 months post-injection [34].

Additional Literature Review:
The literature review could be expanded to provide a more comprehensive discussion of related studies on dopaminergic neuron analysis and AI segmentation. Papers by Haghighi et al. and Barzekar et al. (2023) may provide useful comparisons and insights. Consider incorporating relevant findings from these studies into Table 3 and elsewhere in the discussion.

We appreciate the reviewer’s helpful suggestion to expand the literature review and for pointing out additional relevant studies.

Following this suggestion, we have revised the manuscript to include the study by Haghighi et al. (2023), which proposed a self-supervised learning–based, CNN-backed framework for segmentation and quantification of TH-positive dopaminergic neuron somata. Because this work directly addresses automated quantification of TH-positive neurons, which is the primary focus of the present study, it has been added to the manuscript and incorporated into Table 3 for comparison.

In contrast, Barzekar et al. (2023) reported an automated deep learning–based approach for segmentation of substantia nigra subregions rather than for direct quantification of TH-positive neuronal cell bodies. As the primary aim of the present study is to evaluate the accuracy and robustness of TH-positive neuron counting, region-level segmentation was performed manually and automated regional segmentation was outside the scope of this work. For this reason, Barzekar et al. (2023) was not included in Table 3 or the revised manuscript, although we acknowledge its relevance as a complementary approach for defining regions of interest in future studies.

Overall, we have expanded the related works section to better contextualize our study within existing CNN-based and self-supervised learning approaches for dopaminergic neuron analysis, while clearly delineating the scope of comparison based on cell-level quantification.

- Lines 74-76: To our knowledge, multiple CNN-based AIs have been previously reported for enumerating TH-positive cells in the SNc of neurotoxic PD mouse models [23-27]; however, they are yet to be recognized as the gold standard.
- Lines 184-212 in the **Discussion** section: To our knowledge, multiple deep-learning–based approaches have been reported for automated quantification of TH-positive dopaminergic neurons in the mouse SNc [23–27]. The performance metrics of these models are summarized in Table 3. One of these models exhibited 69.0% precision, 79.3% recall, and 73.0% F1-score, which were slightly lower than those of the present study [25]. The remaining models showed high performance, with precision ranging from 86.0–95.3%, recall from 87.8–95.5%, and F1-scores from 88.0–95.3% [23,24,26,27]. These results indicate that CNN-based AI enables automated TH-positive cell counting, which is difficult to achieve using classical image-processing approaches. Most previously reported models were primarily evaluated using neurotoxic PD models, such as 6-hydroxydopamine (6-OHDA) [23] or 1-methyl-4-phenyl-1,2,3,6-tetrahydropyridine (MPTP) models [24,25], which are known to induce large, dose-dependent reductions in TH-positive neurons (approximately 50–90%) [32,33]. More recently, Chen et al. demonstrated a deep-learning–based quantification pipeline validated in both neurotoxic (MPTP) and PFF models [27], indicating that CNN-based approaches can be applied across distinct PD paradigms. In addition, Haghighi et al. also reported a CNN-based quantification framework applied to PD model mice [26], although the specific PD induction method was not explicitly categorized. Notably, α-synuclein PFF models are generally characterized by more gradual and modest reductions in TH-positive neurons compared with neurotoxic models. The present study focused on the α-syn PFFs model, which has been reported to show an approximately 38% decrease in TH-positive cells at 2 months post-injection [34]. Our results demonstrate that the proposed AI-based approach can reliably quantify TH-positive neurons even under conditions of relatively small neuronal loss. Taken together, these findings indicate that the method implemented in this study achieves performance comparable to previously reported CNN-based approaches while being applicable to PD models with varying degrees of neuronal loss. In addition, by leveraging a commercially available, user-friendly AI platform, our approach facilitates efficient training and application of deep-learning–based neuron quantification without requiring specialized programming expertise, thereby lowering the technical barrier for routine use in experimental neuroscience and histopathological analysis.
- **Table 3.** Performance metrics of CNN-based AIs for counting TH-positive cells reported to date.

| **Report** | **Precision** | **Recall** | **F1-score** |
| --- | --- | --- | --- |
| Penttinen AM et al., 2018 | 88.5% | 87.8% | 88.2% |
| Zhao S et al., 2018 | 95.0% | 91.9% | 93.4% |
| Kim D et al., 2023 | 69.0% | 79.3% | 73.0% |
| Haghighi et al., 2023 | 95.3% | 95.5% | 95.3% |
| Chen et al., 2024 | 86.0% | 91.0% | 88.0% |

Training Dataset and Parameters:
Since the study aims to showcase the application of TruAI for dopaminergic neuron counting, it would be valuable to provide the training dataset and parameters used by the algorithm. This transparency will enable the broader scientific community to replicate or adapt these methods for similar applications.

We appreciate the reviewer’s helpful suggestion.

The complete raw datasets of the reference images will be made available by the corresponding author upon reasonable request.

- Lines 397-398: The datasets generated and analyzed in the current study is available from the corresponding author upon reasonable request.

In addition, we have uploaded the trained TruAI neural network file in zenodo (doi: 10.5281/zenodo.18237443), enabling readers to replicate or adapt our analysis pipeline for related applications.

The parameters used in the algorithm are as follows, described in lines 8-14 in the **Supplementary Methods** section:

Network architecture: U-Net

Data sampler: Balanced

Normalization: Local contrast

Loss function: Softmax cross-entropy

Optimizer: Adam

Geometric augmentation: 90-degree rotations and mirroring

Image processing augmentation: None

In the revised manuscript and supplementary material, we have added this information.

- Lines 325-329: The trained TruAI neural network files are uploaded in Zenodo (doi: 10.5281/zenodo.18237443), entitled “neuralnetwork_THcell_mouse.nn,” “neuralnetwork_THcell_marmoset.nn,” and “neuralnetwork_THcell_human.nn.”.

Moreover, we have added additional literature describing network architecture of our model.

- Lines 442-444: 12.  Falk T, Mai D, Bensch R, Çiçek Ö, Abdulkadir A, Marrakchi Y, et al. U-Net: deep learning for cell counting, detection, and morphometry. Nat Methods. 2019;16(1):67-70. doi: 10.1038/s41592-018-0261-2. PMID: 30559429.

4. Limitations of the Study and Overstated Conclusions:

Marmoset Model Data:
The conclusions drawn for the marmoset model appear to be accurate and appropriately supported by the data. However, the analyses of mouse and human samples do not align with the same level of rigor and should be revised accordingly. Please ensure that conclusions are aligned with the data presented.

Thank you for this important comment regarding the rigor of the analyses and the alignment between the data and the conclusions.

For the mouse experiments, we have clarified the sample size in the revised manuscript. The mouse analysis was designed to evaluate the feasibility and robustness of AI-based counting in comparison with conventional manual counting.

Regarding the human data, we fully agree that the conclusions should be interpreted with caution. The absence of a statistically significant difference between PD patients and healthy controls is most likely attributable to the limited number of available sections, which resulted in insufficient sampling coverage across the rostro–caudal axis of the SNc. This limitation has now been clearly stated in the revised manuscript, and the conclusions related to the human analysis have been appropriately softened to reflect this constraint.

In contrast, the conclusions drawn from the marmoset model remain unchanged, as they are supported by independent manual counting by multiple examiners and a rigorously validated analytical framework. Overall, these revisions ensure that conclusions for each species are appropriately aligned with the rigor and scope of the corresponding data.

- Lines 30-44 in the **Abstract** section: Here, we demonstrate that a commercially available convolutional neural network–based artificial intelligence (AI) counting method (TruAI, OLYMPUS, Tokyo, Japan) enables robust and reproducible quantification of TH-positive dopaminergic neurons in mouse, marmoset, and human SNc samples when compared with conventional manual counting. AI-based counting showed a strong correlation with manual counting across mouse, marmoset, and human samples. Good agreement between AI-based and manual counting was observed in mouse and marmoset samples, supporting the applicability of this approach for cross-species quantification of dopaminergic neurons. In the mouse model treated with α-syn preformed fibrils (PFFs), AI-based counting detected a significant reduction in TH-positive neurons consistent with expert manual counting. Non-experts exhibited greater intra-examiner variability than an expert, indicating that the reliability of manual counting depends on experience. Overall, AI-based quantification provides a robust and objective approach for TH-positive cell counting and may improve reproducibility in dopaminergic neuron analysis, particularly for non-expert users and cross-species studies of PD.
- Lines 99-113 in the ***Validation of AI-based counting against manual counting in mouse SNcd samples*** section: To evaluate the applicability of AI-based counting for TH-positive cell quantification, we compared its performance with conventional manual counting by an expert with more than five years of experience (Fig. 1e-h). AI-based counting showed a very strong correlation with expert manual counting (r = 0.997, p < 0.001, Pearson correlation, Fig. 1i), supporting the feasibility of AI-based quantification in mouse SNcd samples. Bland–Altman analysis further demonstrated good agreement between AI-based and manual counting, revealing a small systematic bias in which AI-based counting slightly overestimated TH-positive cell numbers compared with manual counting, with narrow limits of agreement (−29.4 to −1.1 cells; Fig. 1j). Importantly, no trend toward increased differences at higher or lower cell counts was observed, indicating the absence of proportional bias. Together, these results support the quantitative reliability of AI-based counting in mouse SNcd samples. Consistent with manual counting, AI-based counting successfully identified a significant reduction in TH-positive cells in the PFFs group (p < 0.001, Welch's *t*-test, Fig. 1k), demonstrating that AI-based quantification preserves biologically meaningful group differences.
- Lines 129-131: These results suggest that, within this limited repeated-counting experiment, the variability of manual counting can be influenced by the examiner’s experience.
- Lines 164-169: Consistent with manual counting, AI-based counting detected a trend toward a decrease in TH-positive cell density in PD patient samples compared to controls; however, the difference did not reach statistical significance (n.s., Welch’s t-test; Fig. 3h). The analysis was based on a limited number of sections (three sections from 3 PD patients and six sections from 5 control subjects).
- Lines 172-183 in the **Discussion** section: Here, we demonstrated that our AI-based counting method enables robust and reproducible quantification of TH-positive cells in comparison with conventional manual counting across mouse, marmoset, and human samples, within the scope of the datasets analyzed. Notably, while no established method exists for automatically counting TH-positive cells in non-human primates, our study provides the first demonstration of the applicability of AI-based counting to a marmoset PD model, supported by independent manual counting by multiple examiners. These results underscore the robustness of the method in a biologically and technically challenging primate model. In addition, repeated counting experiments indicated that AI-based counting yielded stable results over time, whereas manual counting exhibited examiner-dependent variability under the tested conditions. These findings suggest that AI-based approaches may contribute to improved objectivity and reproducibility in dopaminergic neuron quantification.
- Lines 225-230 in the **Discussion** section: In conclusion, our AI-based counting method provides a reliable and objective approach for quantifying TH-positive cells in PD model animals. While further validation using larger and more diverse human datasets will be required, this method represents a practical alternative to conventional manual counting and has the potential to facilitate quantitative and reproducible analyses of dopaminergic neuron populations in experimental studies of PD.

TH Cell Counting vs. TH Loss:
The data in the manuscript convincingly demonstrate the ability of TruAI to count TH cells in different species. However, the authors’ claim that they have demonstrated the ability of the software to examine TH cell loss in the PD samples (animal models and human tissues) is not sufficiently supported. This conclusion requires additional evidence and analysis.

We thank the reviewer for this important comment.

We agree that the primary strength of this study is the demonstration of reliable and biologically consistent TH-positive cell counting across species. To address the reviewer’s concern, we have revised the manuscript to clearly distinguish between cell counting accuracy (mouse, marmoset, and human) and assessment of TH cell loss (mouse and human), ensuring that all conclusions are fully supported by the presented data.

While the mouse dataset was used primarily for validating AI-based counting accuracy, the PFF model also allowed detection of TH-positive cell loss, which was consistently observed by both AI-based and expert manual counting.

In contrast, the marmoset and human analyses were intended to validate the applicability of AI-based counting across species. For these datasets, correlation analyses (marmoset and human) and agreement analysis (marmoset) with multiple examiners were performed.

Regarding the human TH-positive cell loss analysis, the absence of a statistically significant difference between PD patients and healthy controls is most likely attributable to the limited number of available sections, which resulted in insufficient sampling coverage across the rostro–caudal axis of the SNc. This limitation has now been clearly stated in the revised manuscript, and conclusions related to the human TH-positive cell loss have been appropriately softened.

These revisions ensure that all conclusions regarding TH cell loss are appropriately aligned with the scope and rigor of the presented data.

- Lines 110-113 in the ***Validation of AI-based counting against manual counting in mouse SNcd samples*** section: Consistent with manual counting, AI-based counting successfully identified a significant reduction in TH-positive cells in the PFFs group (p < 0.001, Welch's *t*-test, Fig. 1k), demonstrating that AI-based quantification preserves biologically meaningful group differences.
- Lines 164-169: Consistent with manual counting, AI-based counting detected a trend toward a decrease in TH-positive cell density in PD patient samples compared to controls; however, the difference did not reach statistical significance (n.s., Welch’s t-test; Fig. 3h). The analysis was based on a limited number of sections (three sections from 3 PD patients and six sections from 5 control subjects).

Human PD Sample Size:
The study analyzes only one human PD sample as per methods. With an n=1, it is not statistically sound to make broader claims about the accuracy of the AI’s detection of TH cell loss in human PD tissues. Please explain how statistical analysis (if any) was performed and provide cautionary notes about these limitations in the manuscript.

We appreciate this insightful comment. Regarding the sample size, please refer to the reply to the section “Discrepancies in Sample Details.”

We believe that the absence of a statistically significant difference between PD patients and healthy controls—observed in both AI-based and manual counting—is primarily attributable to the limited number of available sections. This limited sampling likely introduced bias depending on the anatomical level analyzed along the rostro-caudal axis of the SNc. In the revised manuscript, we have clarified this limitation and discussed that the lack of significance likely reflects restricted sampling coverage rather than a limitation of the counting algorithm itself.

- Lines 167-169: The analysis was based on a limited number of sections (three sections from 3 PD patients and six sections from 5 control subjects).
- Lines 233-238 in the **Limitations of the study** section: A limitation of the present study is the restricted number of human sections available for analysis, which resulted in limited sampling coverage along the rostro–caudal axis of the SNc. This constraint likely reduced the statistical power to detect neuronal loss in PD samples, despite the well-established pathology. Future studies using larger cohorts and anatomically systematic sampling will be required to fully validate the applicability of AI-based counting in human PD tissue.

Data Normalization for Statistical Analysis:
Were the data normalized prior to statistical analysis? If so, describe the normalization process and how the data were represented in the analysis. This information is important for reproducibility and transparency.

We appreciate the reviewer’s helpful comment.

Data normalization was applied only to a specific analysis of the human dataset, where variability in tissue size and anatomical sampling could introduce bias.

For the human samples shown in Figure 3h, cell counts were normalized to the area of the corresponding region of interest (ROI), and the data are expressed as cell density (cells/mm²). This normalization was applied to minimize sampling bias due to differences in tissue size. Each data point represents the area-normalized cell density from an individual section.

For other analyses of the human dataset, cell counts were analyzed without area normalization, as comparable anatomical regions and sectioning conditions were used.

We apologize for the insufficient description in the original manuscript and have clarified these points in the revised manuscript.

- Lines 365-367: For human samples, TH-positive cell density was calculated by normalizing cell counts to the area of the corresponding ROI and is expressed as cells/mm².

Null Hypothesis and AI-Based Analysis:
For the AI-based analyses, what was the null hypothesis? Please explicitly discuss the hypothesis tested and the conclusions drawn based on the results.

We appreciate the reviewer’s important comment regarding the null hypotheses underlying the AI-based analyses. Rather than explicitly stating null hypotheses in the manuscript, we designed each analysis to address a specific validation question, which is reflected in the Results. Below, we clarify the hypotheses tested and the corresponding conclusions for mouse, marmoset, and human samples, and indicate where the relevant results are described in the revised manuscript.

For mouse samples, the null hypothesis was that AI-based counting does not provide measurements equivalent to expert manual counting. This was tested using correlation analysis, Bland–Altman agreement analysis, and group comparisons between PBS and PFFs mice. The null hypothesis was rejected, as AI-based counting showed strong correlation with expert manual counting, good agreement with a small systematic bias and narrow limits of agreement, and successfully detected biologically meaningful differences between disease and control groups.

For marmoset samples, where no established expert reference exists, the null hypothesis was that AI-based counting does not agree with independent manual counting performed by multiple examiners. This was evaluated using correlation analysis and Bland–Altman analysis based on the averaged manual counts from three independent examiners. The results demonstrated strong correlations and overall good agreement with only a minor trend in the differences, supporting rejection of the null hypothesis and indicating that AI-based counting provides quantitatively reliable measurements in a non-human primate model.

For human samples, the null hypothesis was that AI-based counting does not produce results consistent with manual counting or preserve biologically relevant trends observed in human PD pathology. Due to the limited number of available human sections and the absence of an established reference standard, agreement analysis was not used as a primary quantitative validation. Nevertheless, the null hypothesis was not supported, as AI-based counting showed strong correlations with manual counting by independent examiners and reproduced the same directional trend toward reduced TH-positive cell density in PD samples as manual counting, within the stated limitations.

Based on these results, we conclude that AI-based counting provides reliable and reproducible quantification of TH-positive cells in mouse and marmoset samples and demonstrates applicability to human samples within the scope of the available datasets.

In addition, we corrected an error in the description of the statistical tests. Instances in which Student’s t-test had been incorrectly stated were revised to Welch’s t-test, which was consistently used throughout the analyses.

- Lines 372-374: Welch’s *t*-test was used to compare TH-positive cell counts between the mouse PBS and PFFs groups, and TH-positive cell density in the SNc between human control and PD patients.

We also added Bland–Altman agreement analyses for the mouse and marmoset datasets to more explicitly assess agreement between AI-based and manual counting methods.

- Lines 368-372: Agreement between AI-based and manual counting was further evaluated using Bland–Altman analysis, in which the difference between the two methods (Manual − AI) was plotted against their mean. The mean difference (bias) and the 95% limits of agreement were calculated as the mean difference ± 1.96 standard deviations.
- Lines 103-108: Bland–Altman analysis further demonstrated good agreement between AI-based and manual counting, revealing a small systematic bias in which AI-based counting slightly overestimated TH-positive cell numbers compared with manual counting, with narrow limits of agreement (−29.4 to −1.1 cells; Fig. 1j). Importantly, no trend toward increased differences at higher or lower cell counts was observed, indicating the absence of proportional bias.
- Fig. 1j

- Lines 141-146: Bland–Altman analysis demonstrated good agreement between AI-based counting and the averaged manual counts obtained from three independent examiners, with a small systematic bias and narrow limits of agreement (−23.8 to +8.8 cells; Fig. 2h). Differences between the two methods were largely consistent across the range of cell counts, although a minor trend toward more negative differences at higher values was noted.
- Fig. 2h

The title can be improved and a more suitable title would be something like “Evaluation of a Commercial AI-Assisted Cell Counting Software TruAI for Dopaminergic Neuron Counting across species”

We appreciate the reviewer’s helpful suggestion, and the title has been changed to “**Evaluation of a Commercial AI-Assisted Cell Counting Software for Dopaminergic Neurons across species**.”

In summary, while the study demonstrates potential applications of TruAI, improvements in methodology, additional analyses, and consistency in reporting are necessary to fully support the conclusions. This will enhance the impact and utility of the work for the scientific community
