# Supplementary material for: Evaluation of a commercial AI-assisted cell counting software for dopaminergic neurons across species
Source: PLoS One. 2026 Mar 17;21(3):e0344621. doi: 10.1371/journal.pone.0344621 (PMC12994822; doi:10.1371/journal.pone.0344621)
Supplement: Attachment — Submitted filename: Response_to_Reviewers_auresp_2.docx [file pone.0344621.s003.docx]

Reviewer #2: Thank you for thoroughly addressing all the comments. I understand that it is painful to address the reviewers comments but I hope this will make the manuscript stronger and will receive credits in the long run.

I have some very minor comments to better understand,
1.Were reference images also incorporated in counting the cells, if not, why was that route chosen? Better to have counts from all images considering there is limited images in the study which has been highlighted.

Thank you for this comment. The reference images were not incorporated into the cell counting analysis in order to maintain an independent and unbiased evaluation of neural network performance. Previous reports (Lones MA et al. Patterns. 2024) showed that including evaluation images in training would introduce data leakage and could overestimate the performance by allowing the network to memorize image-specific features. Instead, we trained the network using images acquired under identical experimental conditions. This approach follows established best practices in machine learning and ensures reliable and generalizable performance assessment. We have added a literature about data leakage and included the clarification in the revised manuscript.

Lines 326-328: These reference images were used exclusively for neural network training and were not included in the cell counting analysis to ensure an unbiased assessment of neural network performance and to prevent data leakage [45].

Lines 563-564: 45. Lones MA. Avoiding common machine learning pitfalls. Patterns (N Y). 2024;5(10):101046. doi: 10.1016/j.patter.2024.101046. PMID: 39569205.

2. Under "AI-based counting of TH-positive cells in mouse, marmoset, and human samples" it still says 6 human samples but "Human Samples" in methods says 9. Please cross check.

We thank the reviewer for pointing this out. However, we could not identify any sentence referring to “6 human samples” in the section entitled "AI-based counting of TH-positive cells in mouse, marmoset, and human samples" (lines 305-330) of the revised manuscript (version 1).

As correctly noted, a total of 9 sections (3 sections from 3 PD patients and 6 sections from 5 controls) were used for cell counting. This has been described in the section entitled “Human Samples” (lines 302-303) of the revised manuscript as follows: “3 sections from 3 PD patients and 6 sections from 5 controls were used for cell counting.”

In the original manuscript, there was a statement in lines 242-243 indicating that “6 human (1 PD and 5 control) images were selected as reference images.” However, this refers to reference images used for neural network training, not to the sections used for cell counting. In the revised manuscript (version 1), we have clarified this point in line 301 as follows: “1 section from 1 PD patient and 5 sections from 3 controls were used as reference images.”

Importantly, the number of human sections used for cell counting has remained consistent throughout the original and revised versions of the manuscript. Specifically, 3 sections from 3 PD patients and 6 sections from 5 control subjects (total 9 sections) were used for cell counting, and this number has not been changed during revision process. The revisions were made solely to improve clarity and explicitly distinguish between sections and subjects, as well as between reference images and analyzed sections, in order to prevent potential misunderstanding.

We hope this clarification resolves any misunderstanding regarding the human sample size. If the reviewer is referring to a specific sentence indicating a discrepancy, we would greatly appreciate it if they could specify the corresponding line number in the revised manuscript.

3. I didn't see adding Cellpose model as a reference and addressing why this is better than that considering it is the state of art model for cell counting. Additionally, can the authors please add how the model can be made better, for e.g incorporating automated segmentation models for SN as has been shown in some recent manuscripts. That might provide the company to make their workflow and software better for future users.

We thank the reviewer for this important suggestion. We did not intend to claim that our AI-based approach outperforms Cellpose or related pipeline reported by Chen et al. (reference 27). Instead, our primary objective was to demonstrate that a commercially available, user-friendly AI platform can be effectively trained to reliably quantify TH-positive neurons across different PD models, including the α-synuclein PFF model, which exhibits relatively modest neuronal loss.

We agree that incorporating automated segmentation models including Cellpose, represents an important direction for further improving accuracy and automation. Such integration could enhance robustness, particularly in datasets with complex histopathological variability. We have added the Cellpose paper (Stringer et al., reference 35) and included a statement in the revised manuscript acknowledging these potential improvements and future directions.

Lines 212-216: Recent advances in generalizable segmentation frameworks, such as Cellpose [35], have improved the accuracy and robustness of automated cell detection across diverse imaging conditions. Integration of such advanced segmentation approaches may further enhance the accuracy and scalability of AI-based quantification of TH-positive neurons in future applications.

Lines 525-527: 35. Stringer C, Wang T, Michaelos M, Pachitariu M. Cellpose: a generalist algorithm for cellular segmentation. Nat Methods. 2021;18(1):100-106. doi: 10.1038/s41592-020-01018-x. PMID: 33318659.
